# NaTech triggered by lightning: Novel insights from past events in the process industry

**DOI:** 10.1016/j.heliyon.2024.e31610

**Published:** 2024-05-21

**Authors:** David Javier Castro Rodriguez, Joseph Mietkiewicz, Morena Vitale, Gabriele Baldissone, Antonello A. Barresi, Micaela Demichela

**Affiliations:** a Department of Applied Science and Technology, Politecnico di Torino, Corso Duca Degli Abruzzi 24, 10129, Torino, Italy; b Technologic University Dublin Park House Grangegorman 191 North Circular Road, D07 EWV4, Ireland

**Keywords:** Bayesian network, Event tree, Lightning, NaTech, Vulnerability

## Abstract

Lightning strikes, a prominent meteorological event, pose a significant risk of triggering technological disruptions within the process industry. To better understand this phenomenon, an analysis focused on past lightning-triggered events was carried out, examining open-source industrial-accident databases to compile a new NaTech-driven dataset of 689 records. First, an overall quantitative analysis revealed that over 80 % of these events involved incidents or loss of containment. Notably, 83.3 % of them occurred during the spring and summer, indicating a seasonal pattern. Based on the frequency of functional attributes, the chemical and petrochemical macro-sector was the most vulnerable, followed by storage and warehousing. About 40 % of all classifiable events happened on storage equipment, while 21 % happened on electric and electronic devices. Given the lack of valuable information for the principal source of data (NRC), the technological scenarios triggered were characterized using a refined subset of 127 observations, obtained considering the “other sources” of data. Fire scenarios predominated at 56 %; coincidentally, roughly 70 % of all scenarios involved hazardous substances classified as physical hazards. Estimated losses for the available information underscored the adverse consequences of lightning-triggered NaTech events, highlighting their major impact on both safety and the environment. An analysis of the event tree showed the logical path from the lightning strike to the final ignition scenarios (considering a subset of 107 records). This path accounted for 36 % of the classifiable records that directly affected the structure, while more than 50 % of them did not. Bayesian network structures made it possible to get conditional probabilities from the event tree and improved the model by adding attributes for vulnerable equipment and macro-sectors. In order to deal with the uncertain data, algorithms were used to generalize the models that were obtained from smaller subsets of data with more accurate information to the whole dataset. It provides an important additional view of unclassifiable data that otherwise remained in the dark. This novel insight contributes to increase the vulnerability awareness of industrial assets against lightning strikes.

## 1 Introduction

The necessity to strengthen the resilience and adaptive capacity against different natural hazards and disasters is explicit within the framework of Sustainable Development Goals [1]. Moreover, the Sendai Framework for Disaster Risk Reduction 2015–2030 remarked on the importance of the multisectoral mainstreaming multi-hazard approaches and reviewed the coherence with the traditional approaches to achieve the outcome in loss reduction in lives, livelihoods, and health [2]. Thus, the development of holistic risk management approaches in all sectors is an implicit requirement within these international policies.

This contextualization is relevant to the process industry, which is traditionally considered a source of threats, given the hazardousness of its processes and the substances involved [3]. Then, this industrial sector is called upon to ensure sustainable production patterns, reducing the release of hazardous materials to decrease their adverse impacts on human health and the environment [1]. In this context, the process plants can be regarded as complex networks including the infrastructure, the technical components, the physical equipment, the stored raw materials, the flows of materials and energy, the operators, and the organizational elements that interact based on standards, procedures, or instructions [4]. Often, the technological risk community is focused on identifying accident triggers and pathways at the site level while the links to the surrounding territory are neglected [5]. In contrast, process plants are in areas in which natural events can seriously disturb their performance and safety.

In this sense, different natural hazards can affect industrial facilities in several ways, producing direct or indirect loss of containment (LOC) in process equipment that stores or uses hazardous materials causing fires, explosions, or toxic emissions [6]. Therefore, these scenarios may generate a significant impact on the environment and the surrounding population. These kinds of phenomena are known as NaTech, constituting risks posed by chemicals or industrial scenarios triggered by natural factors that involve the release of hazardous materials [7].

NaTech events are generating a significant concern in the industry, and they need the attention of regulatory authorities. Nevertheless, even if in many countries there is a legal framework for the prevention and mitigation of industrial accidents, even with a special address to NaTech events, the mutual impacts between industry and territories are still widely disregarded [8].

In the United States, although the consequences derived from NaTech events are evaluated following the guidelines of the Risk Management Plan (RMP) [9], it is recognized by that these standards, guidance, and codes of practice, address NaTech risk insufficiently [6]. On the other hand, in the European Union, the risks of major process incidents are regulated by the Seveso III Directive (2012/18/EU) [10], which applies to industrial activities that handle or store large quantities of hazardous substances. Currently, the Seveso III Directive requires companies to identify and regularly assess environmental hazards for facility safety [6]. However, operators still consider the environment as a target component for the potential industrial impacts more than a trigger factor [11]. The scenarios related to natural hazards are often classified as “not credible” with likelihoods lower than the order of 10^-^⁶ events/year·site. In contrast, a recent comprehensive analysis of the occurrence of NaTech events, reported an increasing trend of these events occurrence in Europe, during the period 2000–2017, with an average representative value estimated in 3.5·10^−5^ events/year·site [12]. Likewise, a crucial aspect to consider is that industrial sites, which fall outside the scope of the Seveso III Directive due to their hazardous inventory levels, can still be vulnerable to NaTech events. These events have the potential to cause serious harm to human health, and the environment, as well as they may lead to critical accidents.

The NaTech risks can involve loss of containment due to direct infrastructure damages affecting the industrial equipment, damaging safety barriers, or provoking indirect disruption to the auxiliary systems of the utilities. In addition, cascading events may occur establishing a domino effect that can lead to secondary equipment damage. Therefore, the NaTech risk field is interdisciplinary and complex with outcomes scenarios hard to predict and sometimes considered “black swans” [13]. Given this complexity, is relevant to learn from past events, detect signals, and forecast from implicit data yet to be associated or hypothesized. In this line, historical record analysis is essential for evaluating the likelihood of such events, determining the conditional probabilities of natural impact damage to industrial structures, and determining their final scenarios.

Regarding historical analysis, a past study of 232 accident records identified “atmospheric events” accounting for 80 % of the total, with lightning being the most frequent natural factor with 33 % of the occurrences [14]. Moving to more recent studies, a comprehensive study in the process industry examined all 9100 records of NaTech events and discovered that 86 % of them were triggered just by 4 of the 12 categories investigated for natural hazards [12]. The natural factors that practically match the Pareto principle resulted in storms (50.3 %), tropical storms (12.5 %), extreme temperatures (12.2 %), and lightning (11.3 %), in this order. A similar study conducted to understand the patterns and characteristics of Natech events in China confirmed that short-term meteorological events such as rainstorms, lightning, and typhoons are the disasters more likely to cause Natech events in industrial sites [15]. A common characteristic in all the commented previous research is that the natural factors identified as most frequents belong to the meteorological macro-category, and their frequency and severity are expected to be influenced by climate change [16]. Consequently, it is crucial to continue contributing to studies that increase awareness of the vulnerability to these natural factors.

Focus on lightning strikes, presents a critical trade-off based on their frequency and catastrophic consequences in the process industry. Several authors agree that lightning strikes focused on storage and processing activities have been the most common cause of accidents triggered by natural hazards presenting severe consequences [[17], [18], [19], [20]]. For example, a previous study found that lightning-caused accidents can cause severe off-site loss of containment of dangerous substances, weighing between 100 kg and 1000 kg 35 % of the time and more than 1000 kg 38 % of the considered 335 events [21]. Moreover, the high ignition probability of flammable substances following lightning impact (82 %) [18], is higher than the ignition for conventional scenarios reported in the literature [12], which resulted in a relevant number of major fires triggered. Since lightning itself constitutes an ignition source, it is reported as the source of heat for one-third of exterior storage tank fires [19]. In addition, given its recognized potential to cause cascading effects on nearby equipment [22], the fire scenarios following lightning impacts on tanks storing flammable chemicals have reportedly constituted a frequent primary cause of the domino effect [20].

The above discussion highlights that even though the hazards associated with lightning impacts are well known, the protection systems and prevention measures within the process industry may not be enough. Thus, the historical request made by past authors [14,18] about the necessity of conducting more research into the dynamics of direct and indirect lighting effects at chemical facilities is still relevant today, when the NaTech phenomena may be exacerbated by climate change influence.

Hence, the present research develops a historical analysis of NaTech events triggered by lightning strikes in the process industry with novel elements incorporated. It updates the record of such events up to the end of 2022, in comparison to past studies [21] and introduces new comprehensive classifications to improve compatibility with recent studies in the NaTech field and process industry safety [12,18,[23], [24], [25], [26]]. Furthermore, the conceptual updates incorporated into the evolution of international standards regarding lightning protection systems have been introduced as technical criteria [27].

Furthermore, the novelty of this research is also complemented by the incorporation of a modelling subsection that incorporates the updated paradigm related to the propagation pathway of NaTech events [28]. Moreover, Bayesian structures were employed to identify signals, interdependencies, and vulnerabilities among the functional variables and categories analyzed. This process, conceived to face the uncertainty often present in the openly available data, began by refining data sections that included more accurate information. The analysis is then extended to structures with more data that presented missing values typical of the kind of information available in the open-source industrial-accidents databases.

Through these enhancements, the study aimed to raise vulnerability awareness against lightning strikes across all process industry activities. It offers valuable insights and methodological contributions that represent a step toward to strength the resilience of industrial infrastructures against the natural factor of concern.

## 2 Materials and methods

Is duly documented that NaTech accidents are in the range of 1 % and 5 % of the records in the different industrial accident databases [12,14,18]. Then, the methodological cornerstone was the data collection about industrial accidents caused directly or indirectly by a lightning strike. To do that, some steps were conducted, such as the data source selection, the establishment of data retrieval assumptions, the dataset architecture setting associated with the technical criteria definition, and the data quality considerations. The data was mainly retrieved from open-source databases which reported data about past industrial accidents until the end of 2022. In addition, open-literature information was used in some cases to contrast and deepen the information available in the reports. The methodology was integrated under the principle of convergence, from previous research reporting industrial accidents triggered by diverse natural events [12,18,21,[23], [24], [25], [26],^29^]. After the data collection, several analyses and modelling techniques were conducted from diverse perspectives aiming to learn from records to increase vulnerability awareness against lightning strikes.

### 2.1 Data sources consulted

For the compilation of the raw data, several currently available open-source databases on industrial accidents were consulted (ARIA, eMARS, TAD IChemE, eNATECH, NRC, CSB). The FACTS database (Failure and Accidents Technical Information System) which contains several records concerning industrial accidents or near misses, was useless due to the unavailability of the lightning data subset [18]. An overall description of each database used and the data characteristics that they contain is given subsequently.

The ARIA database, overseen by the BARPI (Bureau for Analysis of Industrial Risks and Pollution), encompasses a substantial collection of more than 54,000 records as of the conclusion of 2022. These records encompass a spectrum of accidents, incidents, and near misses that happened within industrial facilities or storage sites, both domestically in France and internationally. Annually, the database sees the incorporation of 1200–1500 new records. The primary source of information documented within this repository primarily emanates from the French Ministry of Ecological Transition and Territorial Cohesion [30].

The eMARS database, which originated from the Major Accident Reporting System (MARS) upon its digitalization, was initially instituted under the umbrella of the EU I Seveso Directive 82/501/EEC in 1982. It has endured over time, aligning with successive iterations of the Seveso Directive that are currently effective. The core objective of eMARS is to facilitate the exchange of insights gathered from accidents and near misses involving hazardous substances that satisfy the criteria for a "major accident" as stipulated in Annex VI of the Seveso III Directive (2012/18/EU) [10]. It is worth noting that while EU Member States are required to report incidents to eMARS when Seveso-designated facilities are involved, reporting non-EU entities is done voluntarily [31].

The Accident Database (TAD) belongs to the Institution of the Chemical Engineers (IChemE), which makes advances in chemical engineering contributions worldwide for the benefit of society, including process safety. The IChemE database was developed in 1997 and contained over 10,500 entries, the last entry was in 2000. It contains data from different sources, including the “Loss Prevention Bulletin”, but limited details are reported; for instance, no details are reported on structural damage suffered by the units where the loss of containment took place, which is extremely important to understand the vulnerability [32].

The eNATECH database, conceived and established by the Joint Research Centre (JRC), serves as a robust framework to bolster the collection of NaTech-related data. Its architecture is designed to encompass the complexity inherent to NaTech events, necessitating an advanced accident representation. The objective of this database is to collect information on NaTech accidents spanning the global spectrum. Furthermore, it facilitates the search and analysis of accident reports, with the primary intent of sharing valuable lessons from these occurrences [33]. Its current repository remains relatively limited in scope; sometimes, the records contained redundantly echo the information already accessible from alternative sources.

The database maintained by the U.S. Chemical Safety and Hazard Investigation Board (CSB) serves as a repository for incident investigation data. The CSB functions as an autonomous federal agency, distinct from regulatory oversight, and it has the responsibility of identifying the fundamental triggers underlying incidents within established industrial installations. This database undergoes a quarterly review process, during which revisions, amendments, or rectifications might be incorporated for events that have been previously documented [34].

The NRC (National Response Center) managed by the U.S. Coast Guard is a federal point of reference for reporting information on oil and chemical spills in the USA. The National Response Center collects records about hazardous releases into the environment anywhere in the United States since 1990 [35]. In the NRC database, it is possible to find short summaries of the most significant industrial accidents. It is important to remark that are registered from voluntary calls of citizens, consequently, the information reported not always is accurate or verified.

### 2.2 Retrieval of data

Since the research scope is focused on identifying vulnerabilities against lightning strokes able to trigger a NaTech event within the process industry as critical infrastructure, then, the following necessary inclusion criteria were adopted to consider the records.

1. The technological event should involve the release (or the potential release) of hazardous, triggered by lightning impact, defined as a single electrical discharge of atmospheric origin between cloud and earth consisting of one or more strokes [27].
2. The type of industrial event should be classified as an accident, incident, or loss of containment event, according to the definitions used by previous authors [12]. For better comprehension, the definitions are inserted below:
  Accident: an event that caused one or more fatalities, and/or permanent major disabilities, and/or heavy financial loss [36].
  Incident: an event that caused considerable harm or loss such as a major health effect or injury, or localized damage to assets and environment, a considerable loss of production, and/or an impact on company reputation [36].
  Loss of containment: event resulting in the release of material [37].
  Near miss: This category considers events that potentially could have resulted in any of the losses defined above, but did not [36].
3. To ensure relevant criteria from the chemical and process industry, the registered technological events occurred in the process industry or eventually in an industrial sector of interest that handles a significant amount of hazardous substances. They should be clustered within the eight industrial macro-sectors used by previous authors [12], which resulted slightly modified from those originally proposed [29]. They are, i) chemical and petrochemical, ii) storage and warehousing, iii) power production, iv) bioprocess, v) water treatment, vi) transportation, vii) pipeline, and viii) manufacturing. Whenever possible, the type of industrial plant was identified.

Once the necessary inclusion criteria are established, the data retrieval process starts by querying databases with single keywords related to the natural hazards under consideration, in line with prior research [12,18,21]. In this instance, keywords such as “lightning,” “thunderstorm,” or “flash” were employed to meet the initial criteria. Retrieving data involves an iterative approach for each consulted database, sometimes necessitating translation of keywords into the database original languages. Subsequently, the most time-consuming phase involves meticulously reviewing each retrieved record summary, to verify remaining criteria, ensuring relevance to the process industry. Particular attention is given to avoiding duplicate entries across databases. To address this, a double-check process is implemented post-data collection, filtering records by date and location. In those cases where the couple date-location appears in more than one case, it is necessary to control the kind of plant, the address, and/or the commercial name of the facility to ensure a unique registration, avoiding duplications or treating events in neighboring facilities as such. In cases of events duplicated, preference is given to the most precise and detailed record complementing the information.

### 2.3 Database architecture

**Fig. 1:**
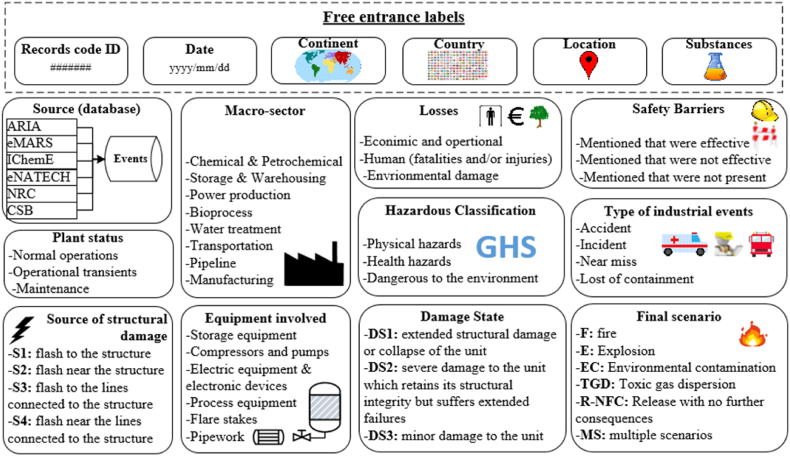
Database structure.

The data retrieved from the different open-source industrial-accidents databases previously described was structured in a NaTech-driven dataset [38] [dataset], following the structure that is shown in Fig. 1.

As can be appreciated, there are labels to classify the information retrieved which was mainly categorical. Many labels are free entrance such as record code ID, date, continent, country, location/city, and substances involved, which correspond with specific descriptive data of the associated record. On the other hand, some labels such as the above-described assumptions (macro-sectors and type of industrial events), or other variables of interest for the classification, require technical definitions for its categorization. Diverse criteria established in the literature were used. The criteria adopted and the diverse categories are described below:

**Plant status**: i) normal operations, ii) maintenance, iii) operational transients (start-up, emergencies, shutdown), according to previous definition [23].

**Source:** Correspond with the raw information originating from open-source industrial databases described in 2.1.

**Equipment involved:** the categories referred to in Renni et al. [21] were slightly modified, homogenizing some items with the categories used by Ricci et al. [25]: i) storage equipment (including warehouses), ii) flare stakes, iii) electric equipment and electronic devices (including instrumentation devices), iv) pipework, v) compressors and pumps (machinery), vi) process equipment.

**Source of structural damage**: i) flash to the structure (S1), ii) flash near the structure (S2), iii) flash to the lines connected to the structure (S3), iv) flash near the lines connected to the structure (S4). Not only the four categories but also the descriptions of what kind of consequences can frequently cause each category were considered, following current international standards [27].

**Damage state**: Since the previously described source of structural damage may cause direct or indirect damages to the equipment able to provoke the LOC, then, three states of structural damages were adopted according to previous authors [21]: i) extended structural damage or collapse of the unit (DS1), ii) severe damage to the unit which retains its structural integrity but suffers extended failures (DS2), iii) Minor damage to the unit (DS3).

**Final scenario:** The categories defined in Ricci et al. [12] were adopted in line with the European Commission report [39]. They are Fire (F), Explosion (E), Toxic Gas Dispersion (TGD), Environmental Contamination (EC), and Release with no further consequences (R–NFC). The combination of two or more of the previously mentioned scenarios was also contemplated as multiple scenarios (MS).

**Safety barriers:** The information was stratified into four categories. i) explicitly recognized they are not present, ii) presence of effective safety barriers, iii) mention of non-effective safety barriers, iv) any reference to safety barriers (unknown).

**Losses:** This label was subdivided into three distinct categories, i) Economic and Operational, ii) Environmental damages, and iii) Human Losses. Their descriptions are offered below:

Economic and operational: the losses were categorized using the same economic strata used by Ricci et al. [26]. An extra subcategory, labeled “not estimated,” was introduced for cases where records lack information about economic losses.

Environmental damage: Environmental damage was sorted into two primary subcategories: “severe environmental damage” and “minor or moderate damage.” These categories were established due to the quality and scope of available information. The classification of severe damage relies on the criteria outlined in point 3, “Immediate Consequences for the Environment,” as detailed in Annex VI of the Seveso III Directive [10]. Similarly, instances of minor or moderate damage refer to situations where environmental impacts were limited or mitigated following the loss of containment (LOC) event. The ambivalence of the categories (severe damage and minor/moderate damage) was adopted in correspondence with the criteria for environmental vulnerability [40].

Human losses: This category was split into “fatalities” and “injured, evacuated, or affected persons”, in line with criteria included in Annex VI of the Seveso III Directive [10].

• Fatalities: This category accounts for events involving the loss of human lifes. It distinguishes between incidents reporting a single fatality and those reporting multiple fatalities [25,26].
• Injured, evacuated, or affected persons: The injured individuals were categorized analogously to the fatalities. Additionally, this category encompasses the count of individuals evacuated due to the NaTech event, along with damage incurred by essential services like drinking water, electricity, gas, or telephone systems.

**Hazardous classification:** This label is directly connected to the free entrance label “substances”. Once every single substance involved in the NaTech events is identified, subsequently the principal categories “physical hazards”, “health hazards”, and “dangerous to the environment” are adopted according to the Globally Harmonized System of Classification and Labelling of Chemicals (GHS) [41]. This classification system for dangerous substances matches with the one used for Seveso establishments referred to in Column 1 Annex I of both Directive 2012/18/EU.

### 2.4 Data quality considerations

As evidenced by previous authors [12,18,24], the information available in the open-source industrial-accidents databases is not homogeneous. Often the information is not precise, or not accurate in describing the event, the failure mode, the structural damage, the presence of barriers, or the severity of the final scenario. Consequently, the possibility of learning from accidents is limited by the quality of data which often is uncertain. Aiming to face this uncertainty by implementing further advanced methods, during the data collection phase, some considerations should be made. For instance, the subcategory “others” was inserted for records in which the information available does not match the predefined technical criteria. In addition, when the lack of information did not allow for the classification of a category, the “unknown” subcategory was added for all labels in the Natech, aiming to underscore the information uncertainty.

### 2.5 Data analysis

#### 2.5.1 Overall analysis

The data analysis was conceived in two principal phases, the overall analysis, and the modelling. The overall analysis aims to provide a clearer characterization of the historical records through a combination of tabular and graphical analyses to explore the diverse labels and subcategories. These analyses encompassed traditional tables, bar, and pie graphs, as well as specialized techniques commonly used in quality control practices such as stratification and control charts. The latest is a notably sensitive method for identifying deviations from randomness. These charts may adopt a three-zones format to demarcate zones of variability around a central line. A process is considered stable when observations span both sides of this central line and remain within control limits, devoid of patterns indicative of non-randomness. This tool was applied following the criteria established in the literature [42].

In a nutshell, First, the origin of records concerning the original data source and its geographical provenance is identified. This step clarifies elements of the data, given the known heterogeneity in the information recorded depending on the different origins of the sources. Second, crucial aspects of the collected information are characterized such as trend, variability, seasonality as well as, the identification of type of industrial events. The third is to estimate the frequency of functional attributes of interest within the Natech-driven dataset to have a first glance at the industrial vulnerability against lightning. Once an initial assessment of functional vulnerability is completed and data limitations have been understood, technological scenarios are characterized. These scenarios are tailored to subsets of data that contain sufficient information aiming its association with other functional attributes. Furthermore, the analysis of consequences is done, based on the available information of the selected subset of data (refer to technical criteron “losses”). Further elaboration on the specific analyses conducted within the overall analysis phase will be exemplified later during the description of the results.

#### 2.5.2 Modelling

##### 2.5.2.1 Event tree analysis

After the characterization of technological scenarios, it is necessary to understand the complexity of the logic events pathways from the triggering natural factor and branches that lead to increasingly specific potential consequences. To this aim, the event tree analysis was implemented which presented several applications for natural hazard modelling [21,43,44]. Furthermore, the inclusion of the new paradigm in the pathway of NaTech [28], also associated with structural intermediate attributes between the initiating factor and the final scenarios, undoubtedly strengthens the identification of vulnerabilities.

For the sake of precision, this technique may be tailored to refined datasets, which means subsets of interest derived from previous steps or with more accurate data within the Natech-driven dataset. Assuming independence between the considered events, the probabilities were calculated considering the relative frequencies from the records in the dataset selected as in equation (1). Then,

(1) $$P\left(n\right)=\frac{n}{N}$$

where:

*P*(*n*) is the probability of the event “*n*”.

“*N*” is the total number of observations within the historical data.

“*n*” is the frequency with which event n occurred within the considered *N*.

Additionally, the conditional probability, written in the form *P*(*n|n–*1) was considered [44]. It consists of the probability of event *n* given that event (*n–*1) has occurred. The probability of any outcome, *P*(*n*), is the product of the probability of an initial event, *P*(1), and all further conditional probabilities, *P*(2*|*1) … *P*(*n|n–*1), leading to that outcome as represented in equation (2).

(2) *P*(*n*) = *P*(1) × *P*(2*|*1) × *P*(3*|*2) × ··· × *P*(*n|n-*1)

##### 2.5.2.2 Bayesian network analysis

Subsequently, starting from the modeled event tree, a Bayesian network structured was mapped [45]. Bayesian networks (BNs) with demonstrated efficacy in the realm of NaTech [[46], [47], [48], [49]], emerged as a versatile framework for predictive modelling and probabilistic reasoning under conditions of uncertainty. Thus, more variables within the NaTech-driven dataset were tested to learn more about the functional vulnerability governed by the conditional interdependencies among attributes.

A Bayesian network can be thought of as an acyclic-directed graph (DAG), where each node represents a random variable, and each arc represents a cause-and-effect link between them. Each node may have different states, and the probability of each state depends on the states of its parent nodes. A conditional probability table (CPT) associated with each node captures this information, specifying the states of probabilities given the states of the parent nodes [46].

Specifically, the Necessary Path Condition (NPC) algorithm was implemented to define the structural framework in the BNs model [50]. The NPC algorithm can estimate parameters even when the data is missing or incomplete. This is particularly useful for the research scope, given the uncertainty that comes with data from open-source databases for industrial accidents. Additionally, the NPC algorithm empowers the user to make informed selections of edges and directions based on causal or phenomenological considerations, in situations where ambiguity may arise from the data. The model parameterization was achieved through the utilization of the Expectation-Maximization (EM) algorithm [51].

The NPC is based on the Peter-Clark (PC) algorithm [52], which uses conditional independent tests to find the structure of the model. The steps of the PC algorithm are as follows: a) A test for (conditional) independence between every pair of represented variables is conducted; b) identification of the skeleton of the graph induced; c) identification of colliders; and d) identification of derived directions. This method helps to identify which links are reliable and which might be weak. By testing different significant levels (α), it is possible to decide which connections in the network are truly supported by the data and avoid including those that might happen by chance. The significant levels of 1 %, 5 %, and 10 % were conventionally chosen given their use in the specific literature [53], ensuring an acceptable level of confidence for the expected results.

For the sake of clarity, a collider is a V-structure, represented as X→Y←Z, which is identified as a specific pattern of conditional independence. This V-structure is characterized by the fact that X and Z are independent unless Y is observed, at which point X and Z become dependent. Therefore, when this configuration of dependencies is observed, it suggests the existence of a collider at Y. The Bayesian Information Criterion (BIC) was also used to show the trade-off between model complexity and how well the model fits the data when BN models were expanded from reduced datasets to larger ones; a higher BIC value is better [54]. Comprehensive details of the algorithms here mentioned can be found in the specific literature [55], while the professional software Hugin 9.3 was used to implement them [56].

## 3 Results and discussion

This section presents the main findings on industrial events caused by lightning strikes within the process industry, using a new NaTech-driven dataset, as the main output of the data collection. This NaTech-driven dataset included 689 records (rows) and 22 columns corresponding with the technical criteria (labels) of interest and has been saved in a public repository [38]. First, a characterization is presented as an overall analysis, describing the records from diverse analytical points of view, such as i) source of data and geographic information, ii) characterization of industrial events, iii) first glance of the industrial vulnerability to lightning strikes, based on frequency analysis of raw data, and iv) technological scenarios characterization. Then, a modeling section (v) to learn from past data is developed. Finally, the novel insights and some lessons learnt are summarized.

### 3.1 Source of data and geographic information

Table 1 shows the number of records retrieved from the different open-source databases, and the percentage that they represent regarding the total of records.

**Table 1:** Records frequency by Source.

Source	Number of records	Percentage (%)
ARIA	114	16.55
CSB	3	0.43
eMars	6	0.87
eNaTech	4	0.58
NRC	562	81.57
Total	689	100

As can be appreciated, most of the records were reported by the American NRC database and in second place by the French database ARIA, which together account for about 98 % of the records analyzed. Therefore, it is not coincidental that an identical percentage of 98 % matches the geographic distribution of records registered in North America and Europe. In particular, the United States and France are the two countries where most records were found, 85 % and 10 %, respectively. In contrast, a minority of events (1.74 %) were reported in facilities from Asia, Africa, or South America.

While NaTech events triggered by lightning strikes are not exclusive to Europe and North America, this observation underscores the global imperative to systematically collect and standardize information about such incidents worldwide.

In terms of the distribution across various sources, the allocation of records aligns with prior research on NaTech accidents [12,26], thereby establishing a foundation of consistency. Specifically, when considering lightning strikes as a triggering factor, the NRC database encompassed approximately 81 % of the records, mirroring closely the percentages documented in earlier lightning-focused studies [21]. Conversely, the tally of records within the ARIA database, previously reported by these same authors at 45 records, has more than doubled over the twelve-year interval following their research, in comparison to the current investigation findings of 114 records.

### 3.2 Characterization of industrial events

#### 3.2.1 Trend of the events

**Fig. 2:**
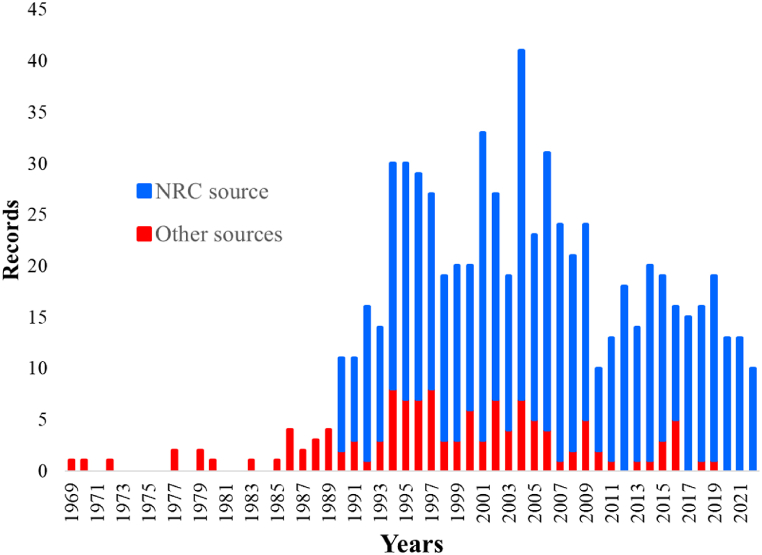
Retrieved records lifespan analysis.

Fig. 2 provides a representation of the record trend over the years, illustrating the lifespan of events. A color convention has been applied to distinguish between records from NRC (blue bars) and other sources (red bars). This assumption is applied consistently hereinafter.

NRC observations dominate the total record count. The temporal trajectory illustrated by the red bars aligns with a period that commenced in the mid-1960s to the 1970s, persisting through to the present day, a span marked by the growth and solidification of the process industry in Europe [4]. On the other hand, blue bars started in 1990 which corresponds with the date that NRC started to record events.

Notably, a sole record is documented before the temporal scope depicted in Fig. 2. This record recounts an explosion within a pyrotechnic production facility in 1866, likely triggered by lightning. Since the considerable temporal remoteness regarding the total records, this record has been excluded from the lifespan analysis, however, it was considered for subsequent analysis of frequency.

While the present research yielded extensive information on industrial events from all sources, the data about natural events is generally confined to acknowledgments of lightning strikes impacting facilities or elements in proximity. Unfortunately, these sources rarely report details essential for characterizing the intensity of these natural events.

#### 3.2.2 Events year variability regarding the source of data

**Fig. 3:**
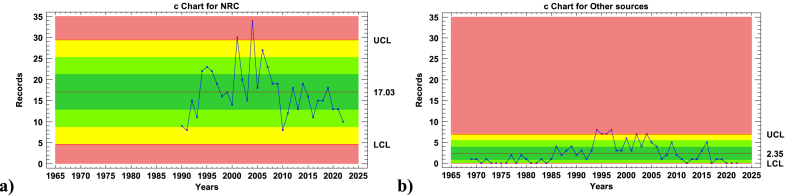
Individual control charts for the records retrieved. **a)** NRC **b)** Other sources.

Given the differences in the number of observations, Fig. 3 provides a more detailed comparison between NRC records and those from other sources. Using control charts set at the same scale serves as a valuable instrument to highlight temporal stability. Furthermore, the graphical analysis yields insights into key statistics of records central tendency and dispersion.

Fig. 3 a) shows a pattern reminiscent of a quasi-steady process. The data points adhere to random variability, except for instances in the years 2001 and 2004 which both go over the upper-control limit line (UCL). These two years stand out as peaks in reported events, recording 30 and 34 occurrences respectively. Across the entire observation period, a mean of roughly 17 lightning-induced events within the process industry annually is discernible in the United States. The range spans from a minimum of 8 to a maximum of 34 events, summing up a spectrum of 26 events which represent the high variability. Notably, post-2010, there's a subtle decline in the number of reported events, with 10 of the subsequent 13 years consistently below the centerline, reflecting the mean value.

Moving to Fig. 3b), a clearer picture of the unstable process emerges. A prominent lack of control is evident over a significant duration. Particularly noteworthy is the span between 1990 and 2010, where 16 out of 20 data points exceed the centerline. This duration witnesses 6 observations surpassing the upper control limit (UCL), while an additional 5 observations cluster in the zones marked B and A above the centerline (bands furthest from the center line within the UCL), indicating excessive variability. Impressively, this timeframe accounts for a substantial 70 % of the records analyzed from “other sources” databases. Within this context, the average occurrence of approximately 2 lightning-triggered events annually within the process industry stands out. Primarily focused on France but encompassing other global locations, the observed variation spans from a minimum of 0 to a maximum of 8 events throughout the analytical period.

#### 3.2.3 Type of industrial events identification

**Fig. 4:**
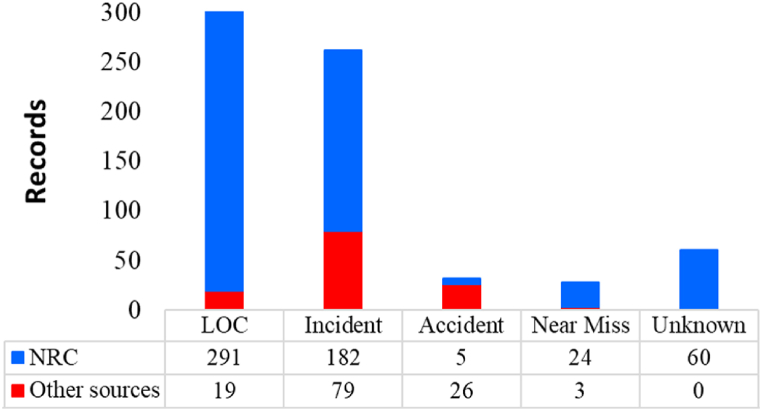
Frequency for type of industrial event.

The classification of industrial events (type) holds paramount significance within the scope of this study. This classification serves as a pivotal determinant in comprehending not only the repercussions of NaTech incidents triggered by lightning strikes but also in separating insights into the incidents reported across the analyzed regions. Fig. 4 clearly illustrates the categories for the event classification that are specified as one of the three inclusion criteria as stated in subsection 2.2 (Retrieval of data).

Upon initial observation of the preceding figure, it becomes evident that the categories of incidents and LOC collectively constitute a substantial majority, accounting for over 80 % (571 records) of the entire compilation of NaTech events reported across all consulted sources. Correspondingly, events leading to fatalities or enduring significant disabilities (accidents) constitute a smaller proportion, around 4 % (31 records) of the overall count. This proportion aligns with the representation of near misses, which counted 27 records of the total.

Regarding the recorded types of industrial events, most of them happened during normal plant operations. In fact, out of the entire dataset of analyzed events, a mere seven instances were reported when the plant was in operational transients. Additionally, just two reports indicated the plant was under maintenance.

However, examining deeper into the graph uncovers noteworthy disparities between the sources. For example, while the NRC accident category comprises less than 1 % (5 events), the same category accounts for roughly 20 % of records from other sources. This stark contrast is astonishing, especially considering that the non-NRC sources exceed accidents by more than fivefold, even though the NRC total record count is five times larger. On the contrary, the LOC category constitutes about 15 % for the other source bars, yet it amounts to over 50 % for NRC. This discrepancy might stem from variations in information reporting and registration regulations within the two principal geographical zones analyzed.

It is crucial to highlight that in tandem with event occurrences, an evaluation of safety barriers was conducted, revealing disheartening findings. Notably, a substantial 92 % of total events lack any reference to safety barriers in their available information. This deficiency includes all records from the NRC source (562). Comparatively, events documented in other sources presented a slightly improved scenario, with about 40 % (51 records) addressing safety barriers. Among these, 21 events acknowledged the presence of effective safety barriers, 20 reported the inefficacy of anticipated safety measures, while merely 10 records explicitly acknowledged the absence of barriers during the disruptive event.

Furthermore, it becomes evident that the NRC category labeled as “unknown” comprises 60 records (as guided by the considerations outlined in section 2.4). In essence, these records exhibit attributes that suggest a NaTech scenario triggered by a lightning strike in the process industry. However, the available information within these records is not enough to identify key details such as fatalities, injuries, material releases, or hazardous situations without notable consequences. This ambiguity may tentatively categorize them as instances of minor LOC or near misses. This interpretation is based on a simple assumption that during the information and registration process, insufficient importance was attributed to capturing aspects involving injuries, fatalities, or significant material releases.

Indeed, most records within the NRC database provided only the minimum necessary elements to confirm that an event was triggered by a lightning strike. Indistinctly, basic additional details, such as the affected equipment, the type of dangerous substance, the nature of the facility, or the ultimate scenario, allowed these records to qualify for inclusion based on the retrieval of data criteria exposed in 2.2.

As previously mentioned in section 2.1, the NRC database compiles reports from observers and voluntary citizens, irrespective of their direct involvement in the facility. Consequently, the reported information is not always verified. This implies the number of reported events is noticeably greater than other sources. Consequently, while the NRC database is abundant in registered records, it aligns with previous research that highlights the “less than ideal” quality of information associated with the records [12,21,24].

#### 3.2.4 Seasonality of the events

Despite the distinctions between NRC records and those from other sources, a significant underlying consistency emerges concerning the seasonality of these events. This issue can be attributed to the shared seasonal patterns between Europe and North America, both of which reside in the northern hemisphere. Consequently, the progression of seasons occurs concurrently in these regions.

**Fig. 5:**
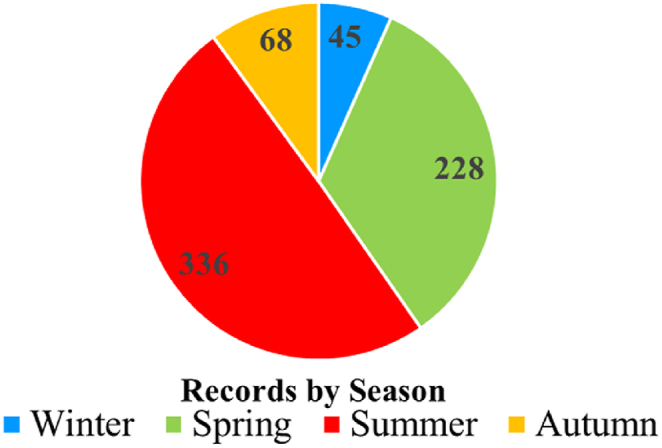
Seasonality of the NaTech triggered by lightning in the process industry, for Europe and North America.

To analyze seasonality, observations from parts of the world other than Europe and North America (13 records), were removed from the dataset. Fig. 5 associates the incidence of NaTech triggered by lightning in the process industry, with the different seasonal fluctuations in the northern hemisphere. The criteria for clustering the observations within each season were centered upon specific date ranges: i) Winter (21 December−20 March), ii) Spring (21 March−20 June), iii) Summer (21 June−22 September), and iv) Autumn (23 September−20 December).

The data illustrates a predominant trend of these events occurring primarily between the spring and summer seasons, accounting for a combined total of 83.3 %. Upon closer examination, the events documented by the NRC displayed a cumulative occurrence rate of approximately 34 % (200 records) during spring and 49 % (287) during summer, while in the case of other sources, the percentages are consistent with a 30 % (27) for spring and 55 % (49) for summer.

Since lightning falls within the meteorological macro-category [12], its occurrence pattern aligns with meteorological variations, thus holding significant meaning. Additionally, owing to the meteorological nature of this phenomenon, particular attention should be paid to its potential susceptibility to the impacts of climate change, specifically in increasing their frequency and severity [16].

This observed seasonality pattern intersects conceptual ideas in the manufacturing field such as inspections and audits, that could be considered for proactive planning. Since previous authors have recognized that lightning protection measures (grounding of equipment, tank shunts, lightning conductors, etc.) in poor condition are not effective in preventing industrial accidents [18], conducting inspections on industrial establishments before the critical periods could be a very convenient practice to ensure that lightning protection measures are well maintained. Even if it seems like an obvious aspect, when contextualized to industrial dynamics the reality may be different.

Contextualizing the planning inspections issue in the Italian major hazard industries, the public system of inspections of industrial establishments subject to the Seveso III Directive finds concrete implementation in the planning and scheduling of inspections in establishments under Legislative Decree 105/2015,^1^ as stated in its article 27. Inspections allow a planned and systematic examination of the technical organizational and safety management systems applied in the establishment, where the competent authorities^2^ must establish annual programs, taking into account some indications including: (a) the hazardousness of the substances present and the processes used, (b) findings of previous inspections, (c) reports, complaints, accidents, near misses, (d) establishment for which the probability of incidents is increased by its geographical location, including the possible escalation events or domino effect, (e) groups of neighboring establishments under major accident hazards, (f) vulnerability of the surrounding area concerning the location of the establishment about possible receptors and pathways of propagation of hazardous substances.

These criteria include in item (d) geographical factors that may make an incident more likely, whereas natural factors might be included as implicit factors in this group. However, there is no further consideration of how these potential factors might change with the seasons. Then, monitoring the public inspection plan for upper-tier Seveso establishments in 2017 as a punctual sample [57], shows that 54 % of 161 establishments inspections were programmed during the spring and summer seasons. Contextualizing this percentage to the lightning issue, in more than half of the establishments audited, the corrective actions that could be detected associated with the lightning protection system, probably will not be implemented in time to avoid events in the most critical seasons. Further research contextualized to real case studies is required to develop these conceptual implications in controlling NaTech events.

### 3.3 Frequency of functional attributes

This subsection aboard two critical attributes to characterize the industrial assets based on the frequency of past events. They are industrial macro-sectors and vulnerable equipment.

#### 3.3.1 Vulnerable industrial macro-sectors

**Fig. 6:**
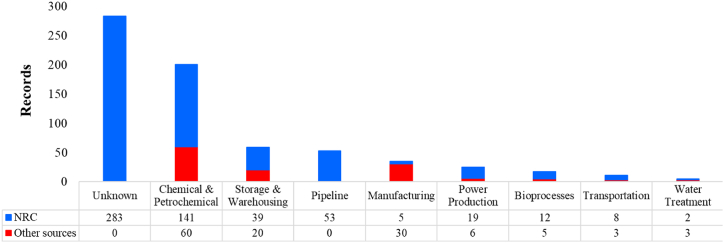
Bar graph for the different industrial macro-sectors affected by lightning strikes.

Fig. 6 illustrates the vulnerability of the different industrial macro-sectors against the NaTech phenomena analyzed.

Setting aside the fact that a significant portion of the events, 41 % (283 events), were categorized as “unknown” due to the information gap in the NRC database, highly valuable insights into the vulnerability of various industrial macro-sectors could be gleaned from the remaining 406 events.

Notably, the chemical and petrochemical sector emerged as the most susceptible to lightning impacts, encompassing roughly 50 % of the known records (201 incidents) across all categories. This observation holds for both NRC and other sources, as evidenced by the proportion of blue and red bars in the chemical and petrochemical bars. This aligns with prior research findings [21], albeit based on a narrower classification and a reduced dataset of 190 records compared to the current study.

Similarly, the storage and warehousing sector represented the second most vulnerable industrial segment, contributing approximately 15 % of the known records. This trend mirrored the balanced contribution of NRC and other sources, each accounting for around 15 % within this category.

As for the pipelines and manufacturing sectors, they made up roughly 13 % and 9 % of the total recorded NaTech incidents, respectively. Unlike the other industrial macro-sectors, these two categories exhibited disparities in the composition of reported events between NRC and other sources. Specifically, the “pipeline” category solely consisted of events from the NRC database (53 out of 53), while the “manufacturing” category displayed a higher proportion from other sources (30 out of 35).

#### 3.3.2 Vulnerable industrial equipment

**Fig. 7:**
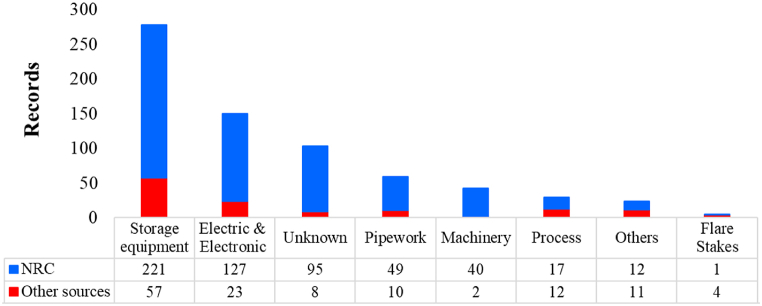
Vulnerable industrial equipment against lightning strikes.

Fig. 7 displays a set of 8 bars representing six distinct categories of industrial items. According to the considerations mentioned in section 2.4, to ensure comprehensive coverage of various individual equipment items that did not neatly align with the pre-established categories, the “others” category has been incorporated into the analysis. In addition, the “unknown” category was also included.

The data reveals a clear pattern where the initial 3 bars are linked to categories boasting more than 100 events each. Leaving aside the “unknown category” due to data quality concerns, primarily stemming from the NRC, if we consider storage equipment as well as electric and electronic devices, there is a total of 428 records, accounting for 62 % of all data.

Specifically, storage equipment emerges as the most vulnerable industrial item, encompassing roughly 40 % of events. This percentage holds not only for the overall events but also when analyzed individually for data originating from the NRC and other sources. This result maintains consistency and aligns with previous research identifying storage equipment as the most frequently impacted element by lightning [20,21,58].

Furthermore, electric equipment and electronic devices display heightened susceptibility to lightning strikes, accounting for over 21 % (150 incidents). This vulnerability arises from induced overvoltage due to electrical discharge, leading to the malfunction of critical components or auxiliary systems, thereby triggering cascading events across various technological scenarios. This proportion remains relatively consistent across the two main components analyzed (NRC and other sources), mirroring the overall trend. Notably, the number of records for this category is nearly three times that reported by Renni et al. using a different dataset [21]. It denotes that more conservative analysis should be considered when implementing lightning protection measures concerning electric equipment and electronic devices, in comparison with past reported data.

The pipework category, with 59 recorded events, ranks next in terms of vulnerability. Although it has slightly more observations compared to earlier research [21], the difference is marginal. The remaining categories exhibit individual contributions below 6 %, displaying heterogeneity in event proportions between NRC and other source components. For instance, the compressors and pumps (denominated as machinery) present 40 to 42 records primarily from the NRC source. On the contrary, the flare stakes category reports 4 to 5 incidents from other sources. An intriguing observation is the diminished event count in the flare stakes category compared to prior reports [21], which could be attributed to the exclusion of the MHIDAS database [59] from our research due to its current unavailability.

### 3.4 Technological scenarios characterization

This analysis focuses on the dataset derived from other sources, which encompasses 127 records. The decision to exclude NRC records was prompted by their lack of crucial information necessary for constructing technological scenarios. This section is divided into two key subsections.

Firstly, the correlation between triggered technological scenarios and the classification of hazardous substances involved is presented. It aims to ascertain the frequency of each cluster of hazardous substances associated with NaTech scenarios, along with identifying the scenarios with the highest incidence.

Complementarily, the severity linked to the different scenarios caused by lightning strikes in the process industry was analyzed considering different categories of losses. It is important to clarify that three entries categorized as near misses within the other-sources dataset, were intentionally excluded from the analysis, then 124 records were used. All the categories used for this analysis were previously defined in section 2.3.

#### 3.4.1 Category of hazardous substances in the triggered scenarios

**Fig. 8:**
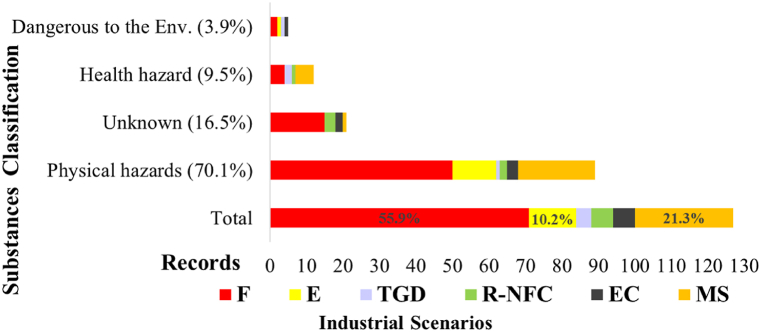
Technological scenarios triggered by lightning vs. classification of substances involved.

Fig. 8 associates the distribution of technological scenarios within the principal categories of physical hazards, health hazards, and dangers to the environment according to the Globally Harmonized System of Classification and Labelling of Chemicals (GHS). In addition, the unknown records were also plotted to compare the pattern for final scenarios for those records in which the available information did not allow the classification of substances involved.

When considering the total number of scenarios, a significant prevalence of red bars is evident across nearly all substance categories. Then, the prominent scenario is Fire (F), accounting for approximately 56 % (71 observations) of the total records (indicated by the total bar at the bottom of Fig. 8). Following Fire, the category with the next highest frequency is multiple scenarios (MS), representing around 21 % (27 records). It is noteworthy that within instances of multiple scenarios, almost always there was at least one scenario involving ignition. This tendency for multiple scenarios is particularly pronounced when the primary scenario is an explosion, accounting for 21 out of 26 records. In third place among the most frequent scenarios is the explosion category (E), constituting approximately 10 % (13 records).

The collective trend highlighted above underscores that scenarios involving ignition—whether single or multiple, immediate, or delayed scenarios—comprise 107 out of 124 records. It was considerably higher than the reported probability of ignition by the lightning strikes, in a precedent study of NaTech events in the process industry with a bigger dataset [12]. Then, results imply being more conservative in terms of managing ignition scenarios.

In terms of the distribution of data by substance category, the prominence of the physical hazards category is conspicuous, accounting for around 70 % (89 records) of the total triggered scenarios. It is important to clarify that the Physical hazards category encompasses different sub-categories including flammable, oxidizers, explosive substances, and more. From these 89 records within the Physical hazards category, a substantial 92 % (82 records) are attributed to flammable substances, of which 63 % (52 records) are specifically associated with different types of hydrocarbons.

Following Physical hazards in terms of frequency is the Unknown substances category, representing 16.5 % (21 records). Notably, the color of bars corresponding to scenarios within the Unknown category suggests a dominance of Fire scenarios. This implies that the unidentified substances involved likely share characteristics aligning them with classifications based on Physical hazards.

#### 3.4.2 Categories of losses involved in the triggered scenarios

**Fig. 9:**
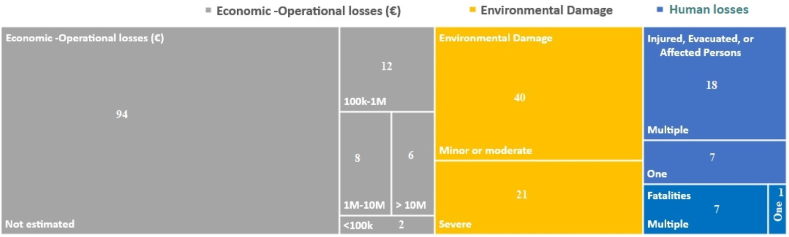
Frequency of different categories of losses within the other sources dataset.

Fig. 9 illustrates the order of distinct subcategories of losses, represented as hierarchical areas within a rectangle, organized from left to right. It is important to note that these subcategories are not mutually exclusive, as an event could match multiple subcategories simultaneously. The number alone within each rectangle corresponds to the observed record frequency.

The largest area within the figure is occupied by economic and operational losses, followed by environmental damage, and finally, human losses; where injured, evacuated, and affected persons, surpass the fatalities.

Paradoxically, within the economic and operational losses subcategory, 94 records pertain to events where losses are not quantified. Even though loss of containment or plant shutdowns should ideally be translated into economic terms in the absence of other losses, the lack of consistency and data homogeneity complicates such evaluations. For cases with available accounting data, significant economic repercussions are evident.

This kind of NaTech often leads to adverse environmental impacts, reported for nearly 50 % of occurrences. Out of these, 35 % were classified as causing severe impacts across various environmental aspects.

Around 20 % of events in the dataset reported injuries, evacuations, or individuals affected by disrupted services due to NaTech incidents. Of these, 72 % affected multiple people. Moreover, events reporting human fatalities accounted for 6.4 % of the total analyzed within the other sources dataset. Notably, out of the observed 8 cases, 7 were instances of multiple fatalities, underscoring the impact of these NaTech events. As can be observed in Fig. 9, most of the quantified losses can potentially be classified as major accident events according to the criteria outlined in Annex VI of the Seveso III Directive issued by European Commission in 2015 [10], even if many of them do not belong to plants regulated by this directive. Table 2 compares the economic and human losses estimated for lightning caused events with the losses estimated for all NaTech events (see “All” column) in the process industry estimated by Ricci et al. [12].

**Table 2:** Comparison of losses for lightning factor and all NaTech events.

Losses	Categories		All	%
Human	Fatalities	One	26	3.8
		Multiple	45	15.5
	Injuries	One	57	12.3
		Multiple	82	21.9
Economic Operational	<100 k €		67	2.9
	100 k€ — 1 M€		33	36.3
	1 M€ — 10 M€		28	28.5
	>10 M€		16	62.5

Although the proportionality presented must be considered with caution since it came from different studies and datasets, they still provide insight into the adverse consequences of the outcomes resulting from NaTech events triggered by lightning within the process industry.

The significant percentages relating to total economic losses, along with human fatalities and injuries (including evacuated and affected people in the case of lightning), emphasize the importance of prioritizing lightning studies and subsequent efforts for its mitigation or strengthening the resilience against this natural factor.

### 3.5 Learning from past events data through modelling

Although the modeling section in the Materials and Methods section was split into the events tree and the Bayesian networks analysis respectively, it will be divided into four subsections during the Results discussion, to better illustrate the flow. Initially, the category of substance with more incidence in NaTech scenarios is deepened, constructing an event tree with a refined dataset form the technological scenarios characterization. Then, a Bayesian network structure was mapped from the event tree analysis to explore the conditional probabilities of functional attributes. Additionally, algorithms to treat the data uncertainty characteristic of open-source databases were implemented, providing a probabilistic meaning to these uncertain data. After, the model was enhanced adding more functional attributes from the other source dataset, to better understand the industrial conditional interdependencies and susceptibilities against lightning strikes. Finally, the insights learnt from the data were extended to the NaTech-driven dataset of 689 records to obtain a second view of the industrial vulnerability of the whole NaTech-driven dataset coping with the uncertain data.

#### 3.5.1 Event tree analysis for a specific dataset

Given the results discussed in subsection 3.4.1, a refined dataset of 107 records corresponding to those finalized with ignition scenarios, was modeled as the event tree. It was built including the labels “sources of structural damage” (*Si*) and “damage state” (*DSj*). These labels serve as intermediate attributes from the initiating event lightning strikes (*LS*) to the final ignition scenarios (*ISk*).

Specifically, the source of structural damages contributed to the identification of the direct or indirect pathway depending on whether primarily structural damage is involved or equipment or utility impairment [28]. According to the standard classification [27], the direct impact on the structure (S1) corresponds to the direct pathway. On the other hand, categories S2, S3, and S4 correspond, respectively, to indirect structural damage or disruptions, whether directly or indirectly affecting the lines that link the structure or auxiliary equipment responsible for its operation. The category of “unknown” was assigned in cases where the sources of structural damage could not be classified with the abbreviation “S?”. The consideration of the label damage state, allows a first view of the pathway through the status of the unit affected, concerning how the lightning impact (source) and its repercussions in the subsequent scenarios triggered.

**Fig. 10:**
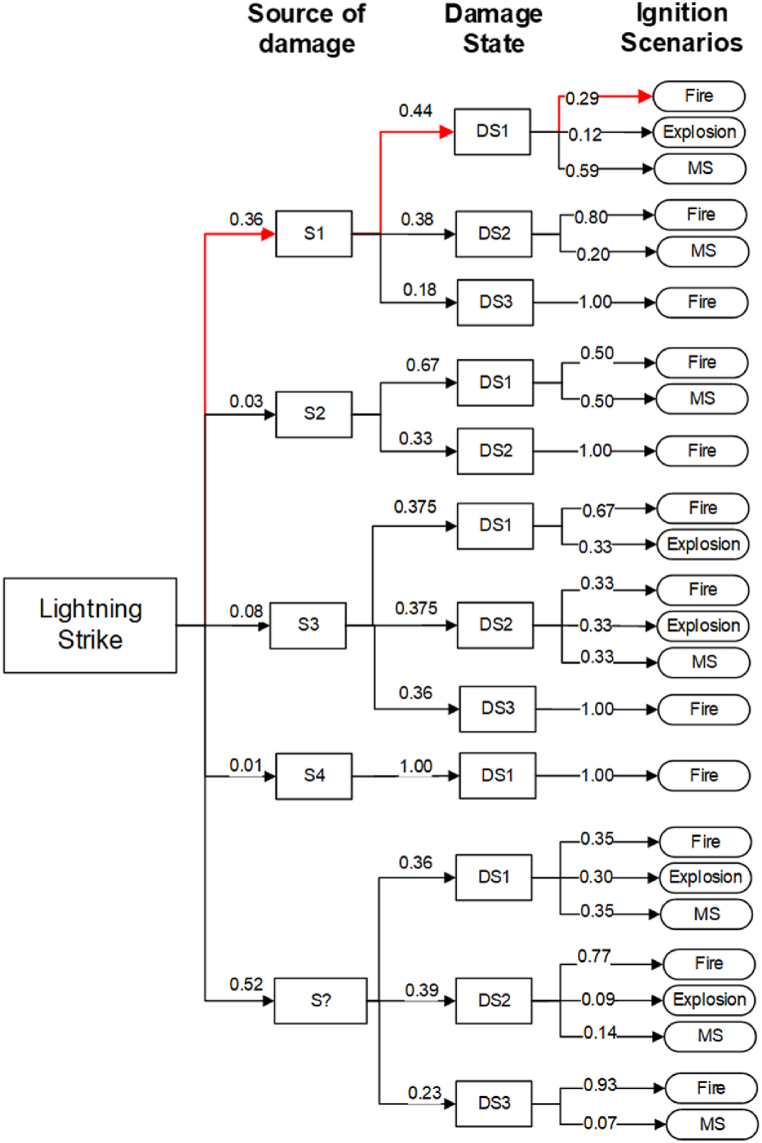
Event tree for the records reporting ignition scenarios (top branch in red to exemplify subsequently calculations). (For interpretation of the references to color in this figure legend, the reader is referred to the Web version of this article.)

Fig. 10 shows the event tree for the sequence (*LS*)→ (*Si*)→(*DSj*)→(*ISk*), from the initiating event, passing through both intermedial events, the “*i*” sources of damage (S1, S2, S3, S4, and S?), and the “*j*” damage states (DS1, DS2, and DS3), finally leading to the considered “*k*” ignition scenarios (F, E, and MS). The probabilities estimated for the considered events within the refined dataset are shown in the connecting arrows of the event tree.

Each branch of the tree represents a distinct sequence of events that could occur. Then, the probability along the branch from the initiating event to the specific outcome was calculated as state equation (3):

(3) (*LS*)→(*Si*)→(*DSj*)→(*ISk*) = P(*LS*) × P (*Si|LS*) × P(*DSj|Si*) × P(*ISk|DSj*)

where:

P(*LS*): Probability of the initiating event. (Since all the records in the analyzed dataset belong to NaTech triggered by lightning that already occurred, then, the probability of the initiating event was considered as certain, P(*LS*) = 1).

P(*Si|LS*): Probability of occurrence of the source of structural damage “*i*” given that the lightning strike occurred.

P(*DSj|Si*): Probability of occurrence of the damage states “*j*” given that the source of structural damage “*i*” has occurred.

P(*ISk|DSj*): Probability of occurrence of the ignition scenarios “*k*” given that the damage states “*j*” has occurred.

For example, calculating the probability in which the specific Fire scenario belonging to the top branch of the event tree (in red in Fig. 10) will be obtained by substituting the probabilities and developing the operation as illustrated in equation (4):

(4) (*LS*)→(*S1*)→(*DS1*)→(*F*)=P(*LS*) × P (*S1|LS*) × P(*DS1|S1*) × P(*F|DS1*)

Substituting,*P*(*LS*) × *P* (*S1|LS*) × *P*(*DS1|S1*) × *P*(*F|DS1*) = 1.00 × 0.36 × 0.44 × 0.29 = 0.045936.

Analogously, other probabilities can be determined. On the other hand, the probabilities of the same final ignition scenarios which are recurrent in diverse branches along the several event three pathways, were calculated by summing the probabilities of all the branches leading to the specific final ignition of interest according to equation (5):

(5) $$P\left(ISk\right)=\sum_{l}P\left(B_{l}\right)$$

where:

*P*(*ISk*) is the probability of the ignition scenario “*k”* occurring (F, E, MS).

*P*(*B*_*l*_) is the probability of branch “*l*” leading to the ignition scenario “*k”*.

Table 3 offers the probabilities of intersection between all the categories within the three variables (source of damage, damage state, and ignition scenarios) used in the event tree analysis (ETA). Probabilities hereinafter are rounded by 10^−2^ which may result in a sum that does not exactly equal one.

**Table 3:** Intersection probabilities from the ETA.

Labels	Category	F	E	MS	Total
**Damage State**	**DS1**	0.15	0.08	0.17	0.40
	**DS2**	0.29	0.03	0.06	0.38
	**DS3**	0.21	0	0.01	0.22
**Source of Damage**	**S1**	0.22	0.02	0.12	0.36
	**S2**	0.02	0	0.01	0.03
	**S3**	0.05	0.02	0.01	0.08
	**S4**	0.01	0	0	0.01
	**Unknown**	0.35	0.07	0.1	0.52

As discerned from Fig. 10 and Table 3, within records where classification was feasible for the source of damage, the S1 emerges as the prevailing pathway, accounting for 36 % of occurrences (65 % of the classifiable records), where explicitly lightning directly stroke the industrial assets. Events following the indirect pathways make up roughly 12 % of the analyzed refined dataset (combining the percentages of S2, S3, and S4). Although constituting a minority concerning the overall occurrences, disregarding these instances could potentially trigger cascades of events with severe consequences.

On the other hand, 52 % of the time it was not possible to classify the source of damage, due to the information available. Nonetheless, some insights can be gathered from the damage states and ignition scenarios. Regarding the issue of the scenarios, the predominant scenarios consistently involve fire (F), as previously discussed in section 3.4.1. Among these, categories S1 and Unknown contribute most significantly to fire scenarios in terms of the source of damage (see Table 3). This pattern suggests that many instances lacking an explicit source of damage classification likely belong to the category of direct structural impact (S1). In the same way, from Fig. 10 it can be elucidated that S1 is responsible for more than 70 % of the collapses and severe damages to the industrial units (DS1 and DS2) in the known records. The high number of events with DS1 and DS2 states within the unknown records suggests that many of these might belong to the direct lightning strike pathway.

Regardless of the source of damage, the damage state is reasonably balanced across three categories of final scenarios. Specifically, unit collapse (DS1) accounts for 40 %, severe unit damage (DS2) comprises 38 %, and minor damage (DS3) represents 22 %. However, focused analysis reveals that when DS1 occurs, the final ignition scenarios tend to be more similar in percentages. This makes sense considering the release states associated with the damage states in Renni et al. (2010), where DS1 is associated with the possible instantaneous loss of the entire inventory. Thus, various types of ignition scenarios can occur and combine from the energy provided by the electric discharge depending on the substance involved. On the other hand, scenarios linked to DS2 and DS3 states, characterized by non-sudden release states, primarily tend to result in fires.

#### 3.5.2 Bayesian network mapping from event tree and subsequent analysis

**Fig. 11:**
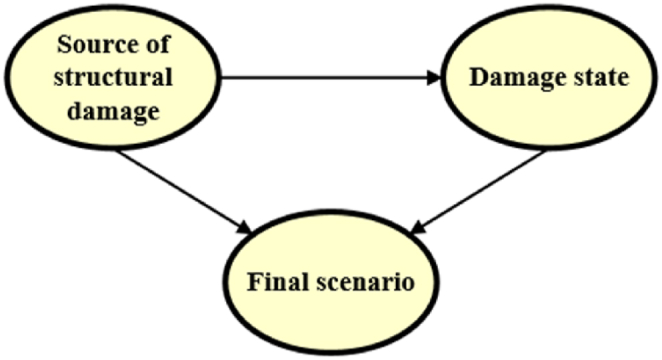
Bayesian Network structure for ignition scenarios in the refined dataset, mapped from the event tree.

To gain deeper insights into the data in the refined dataset, the event tree depicted in Fig. 10 was mapped into the Bayesian network structure shown in Fig. 11. The direction of the arrows in the Bayesian structure, determines the causal relationship between the nodes.

First, conditional probabilities for each node of the model in Fig. 11 were calculated. Second, using the same refined dataset, the conditional probabilities were recalculated by applying the EM algorithm, treating as missing values the “unknown” categories within the “source of structural damage”. Third, this algorithm to cope with the uncertainty was iteratively applied to the extended dataset of 689 records. The conditional probability tables (CPT), comprising both the refined and the extended dataset, are presented in Table 4, in addition, it includes the Bayesian Information Criterion (BIC) corresponding in any case.

**Table 4:**
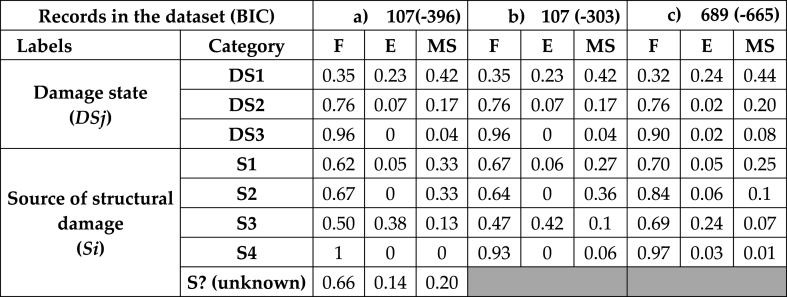
CPTs for the ignition scenarios given “damage states” and “source of damages” for a) refined dataset including S?; b) refined dataset treating uncertainty; c) extended NaTech-driven dataset treating uncertainty.

The present discussion focuses on the utility of techniques to deal with data uncertainty, rather than delving into the specifics of the most probable conditional scenarios. Starting the discussion on the refined dataset (107 records), for the *DSj* variable, since there was no missing data (unknown), the conditional probabilities remained unchanged before and after addressing the uncertainty (refer to Table 4 sections a) and b) focusing on *DSj*). Moving on to the *Si* variable, the conditional probabilities for its categories were numerically consistent with those in the presence of uncertainty. Additionally, the BIC value for the second model was found to demonstrate a better fit in comparison with the initial model, which included unknown values (−396<−303).

Therefore, the expected frequencies of NaTech for *Si* after addressing the uncertainties were recalculated for this refined dataset: P(S1) = 0.74, P(S2) = 0.06, P(S3) = 0.18, and P(S4) = 0.02. It is evident that the probabilities nearly double in every scenario. In particular, the direct pathway for lightning damage to the structure (S1) goes from 36 to 74 % of events, which fits with the subtle pattern we talked about earlier and suggests that unknown records might mostly come from direct pathway events.

The outputs of the second model using the refined dataset were compared with those of the model using all the records in the NaTech-driven dataset. As can be observed, the BIC value for the extended model shows a lower fit compared with the reduced dataset (−665<−303). Previous sessions extensively discussed the high number of unknown records related to information reporting in different geographic zones, which may contribute to the extended dataset complexity. The higher complexity of the dataset may need the inclusion of more variables into the model to increase the fit goodness.

Despite the issue with the BIC value, the conditional probabilities for the same categories in both datasets are largely consistent. In general, fire emerges as the most likely scenario across various damage sources. The most notable variations pertain to the increase in fire probability in the extended dataset for S2 and S3, which change from 0.64 to 0.84 for S2 and from 0.47 to 0.69 for S3, respectively. Consequently, variations in E and MS for the same categories take place. The limited occurrences of S4 in the extended NaTech-driven dataset contribute to the certainty associated with S4.

Similarly, the expected relative frequencies for *Si* were recalculated for the extended dataset: P(S1) = 0.88, P(S2) = 0.05, P(S3) = 0.06, and P(S4) = 0.01. The direct pathway predominates here even more than for the reduced dataset. On the other hand, within the categories associated with the indirect pathway, S2 and S4 remain almost identical in percentiles for both datasets, while S3 experiences a notable reduction in percentile from 0.18 in the reduced dataset to 0.06 in the extended dataset.

#### 3.5.3 Bayesian network model generalization to “other source” dataset

**Fig. 12:**
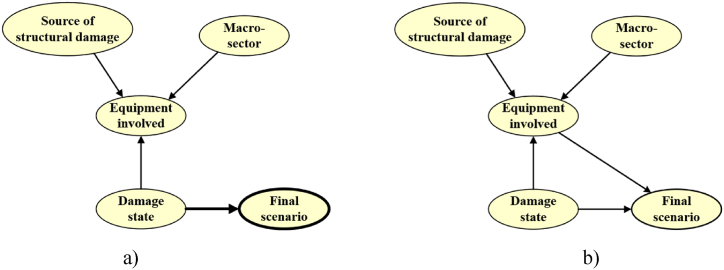
Bayesian Network model learned with NPC algorithm: **a)** level of significance of 1 % and 5 %, **b)** level of significance of 10 %.

The Bayesian network model discussed above was expanded, including two more variables. The functional attributes “macro-sectors” and “equipment,” discussed in section 3.3, were included in the Bayesian structure using the “other sources dataset,” which encompasses 127 records. Its inclusion aims to explore hidden signals of these functional attributes within the model for industrial vulnerability against lightning strikes. The BN structures generated via the NPC algorithm are shown in Fig. 12.

From the structure analysis, it can be appreciated that “equipment involved” is correlated to most of the other variables (with all the variables if a 10 % significance level is selected), identifying it as the key variable in the model. On the other hand, the other variable, “macro-sector,” is independent of the “final scenario” if the equipment is not chosen, making it impossible to get information about the final scenarios only by looking at a macro-sector.

The structure in Fig. 12 a) remains identical by setting the level of significance (α) at 1 % and 5 %, while the direction from “damage state” to “final scenario” (in bold) was chosen based on causal considerations done from the phenomenological pathway. Moving to the structure (Fig. 12 b), when the statistical significance is set at 10 %, the structure remains quite like the previously commented structure, but connections with the node “final scenario” are truly supported by the data. The BIC value of this specific model was −7955. It is important to note that the data analysis in this new model including two more functional attributes vulnerability comports a dataset with just 127 observations which can limit the robustness of some statistics.

**Fig. 13:**
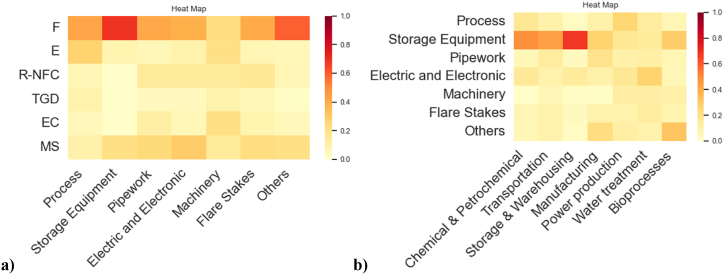
Heatmaps for CPTs other sources dataset: **a)** Final scenario/Equipment involved, **b)** Equipment involved/Macro-sector.

Fig. 13 a) and b) show the heatmaps for the conditional probabilities involving the variables introduced, illustrating interesting conditional susceptibilities not only between the industrial equipment with the final scenarios but also with the industrial macro-sectors. The heatmaps offer an intuitive visual picture of the conditional probabilities represented in Table 5 and Table 6, which were obtained from the model in Fig. 12 b).

**Table 5:** CPT for Final scenario/Equipment involved (other sources dataset).

Equipment		Process	Storage Equipment	Pipework	Electric & Electronic	Machinery	Flare Stakes	Others
**Scenario**	**F**	0.42	0.67	0.41	0.38	0.22	0.4	0.59
	**E**	0.27	0.08	0.06	0.09	0.2	0.07	0.06
	**R–NFC**	0.07	0.01	0.14	0.14	0.14	0.16	0.07
	**TGD**	0.09	0.02	0.04	0.04	0.09	0.06	0.02
	**EC**	0.05	0.01	0.11	0.06	0.21	0.08	0.05
	**MS**	0.1	0.21	0.24	0.29	0.14	0.22	0.21

**Table 6:** CPT for Equipment involved/Macro-sector (other sources dataset).

Macro-sector		Chemical & Petrochemical	Transportation	Storage & Warehousing	Manufacturing	Power Production	Water Treatment	Bioprocesses
**Equipment**	**Process**	0.16	0.10	0.03	0.10	0.26	0.13	0.07
	**Storage Equipment**	0.49	0.43	0.68	0.27	0.16	0.14	0.29
	**Pipework**	0.06	0.13	0.04	0.19	0.10	0.11	0.07
	**Electric & Electronic**	0.15	0.09	0.14	0.10	0.16	0.27	0.06
	**Machinery**	0.01	0.07	0.03	0.02	0.12	0.12	0.10
	**Flare Stakes**	0.07	0.09	0.04	0.09	0.09	0.13	0.08
	**Others**	0.06	0.09	0.03	0.22	0.11	0.09	0.32

* No entries were found in the dataset “other sources” for the pipeline macro-sector.

Fig. 13 a) shows that each equipment category may be associated with Fire as the predominant conditional final scenario. These findings align with the broader discussion in section 3.4.1. Across all equipment categories, the fractions attributed to the fire scenario ranged from 0.42 to 0.67. Conversely, the least conditional probable scenario, Toxic gas dispersion (TGD), ranges from 0.02 to 0.09 percent among the different equipment categories. Moving to Fig. 13 b), it is noticeable that given the sectors like chemical and petrochemical, transportation, and storage and warehousing, the storage equipment category stands out as the most vulnerable component, with prevalence proportions ranging between 0.43 and 0.69 of recorded events in the dataset. On the other hand, storage equipment exhibits more diverse percentages among sectors like manufacturing, power production, water treatment, and bioprocesses. In general, electric equipment and electronic devices are the category gathering the second importance within the vulnerable equipment, considering the values accumulated across all the macro-sectors.

The information generated is useful to rate vulnerable equipment depending on the macro-sector to which the facility belongs. From this, information about the final scenario probabilities can be elucidated. It could be crossed with the characteristics of the dangerous substances detained in process plants. Furthermore, it can be associated with both, the meteorological and environmental information related to the plant location, allowing the objective assessment of the NaTech vulnerability of industrial infrastructure, against lightning impacts, considering from a holistic point of view, the industrial context particularities as discussed in previous studies [60]. The model BIC value was −7955. It is important to note that the data analysis in this new model which includes industrial vulnerability variables (macro-sector and equipment), comports a dataset with 127 observations (other sources dataset) which can limit the robustness of some statistics.

#### 3.5.4 Bayesian network model generalization to the extended NaTech-driven dataset

The analysis was generalized to the extended dataset (689 records), which has numerous missing values as discussed previously. Similar to the previous analysis, the NPC algorithm was employed for structure learning, while the EM algorithm was used for parameter learning. In this case, the Bayesian structure obtained identically aligns with the one depicted in Fig. 12 b), using levels of significance of 5 % and 10 %.

**Fig. 14:**
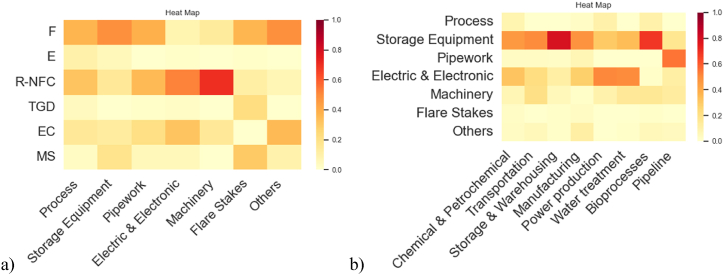
Heatmaps for CPTs extended dataset: a) Final scenario/Equipment involved, b) Equipment involved/Macro-sector.

The BIC value for this model using the extended dataset offers a worse fit to the data than the “other sources” dataset (−13830 < −7955). Since the extended model is derived from a wider dataset with more imprecise data, it might suggest the necessity of introducing more variables to improve the fit. Analogously to the results with the reduced dataset, further details about the industrial vulnerabilities are presented throughout the heatmaps in Fig. 14, while the conditional probabilities represented in the heatmaps are offered in Table 7, Table 8.

**Table 7:** CPT for Final scenario/Equipment involved (extended dataset).

Equipment		Process	Storage Equipment	Pipework	Electric & Electronic	Machinery	Flare Stakes	Others
**Scenario**	**F**	0.37	0.49	0.38	0.08	0.14	0.36	0.49
	**E**	0.10	0.05	0.00	0.01	0.00	0.01	0.00
	**R–NFC**	0.32	0.15	0.35	0.52	0.70	0.11	0.07
	**TGD**	0.04	0.00	0.01	0.02	0.00	0.22	0.00
	**EC**	0.15	0.13	0.21	0.32	0.15	0.00	0.35
	**MS**	0.03	0.18	0.05	0.05	0.00	0.30	0.09

**Table 8:** CPT for Equipment involved/Macro-sector (extended dataset).

Macro-sector		Chemical & Petrochemical	Transportation	Storage & Warehousing	Manufacturing	Power Production	Water Treatment	Bioprocesses	Pipeline
**Equipment**	**Process**	0.08	0	0	0.02	0.1	0	0.1	0
	**Storage Equipment**	0.46	0.5	0.79	0.48	0.3	0.34	0.68	0.17
	**Pipework**	0.03	0.03	0.02	0.07	0	0	0	0.55
	**Electric & Electronic**	0.32	0.22	0.11	0.28	0.51	0.5	0.01	0.11
	**Machinery**	0.07	0.02	0.05	0.01	0.09	0.15	0.16	0.13
	**Flare Stakes**	0.02	0	0.01	0.03	0	0	0.01	0
	**Others**	0.03	0.05	0.01	0.11	0	0.01	0.05	0.04

Upon examination of Fig. 14 a), the predominant final scenarios given the equipment were Fire and R–NFC (release with no further consequences). Regarding the fire scenario, the results align with that obtained for the other sources dataset (see Fig. 13 a). Conversely, R–NFC emerged critically when the NRC records were included (extended dataset), as well as the EC scenario noted and increased. These two rows darken compared to Fig. 13 a). Interestingly, in a study examining the occurrence of NaTech incidents in the process industry across twelve categories of natural hazards, it was noted that the two scenarios with the highest incidence were like the findings of this research, with NRC records contributing to nearly 90 % of the data.

Moving to Fig. 14 b), storage tanks remain clearly as the critical equipment implicated. However, in sectors such as power production and water treatment, there is a marked involvement of electrical equipment and electronic devices more pronounced than for the smaller dataset. Within this new analysis, the category of pipes emerges as critical in the pipeline macro-sector, it can be noted that, on the contrary, in Fig. 13b), no entries were found for the pipeline macro-sector (see note in Table 6).

**Fig. 15:**
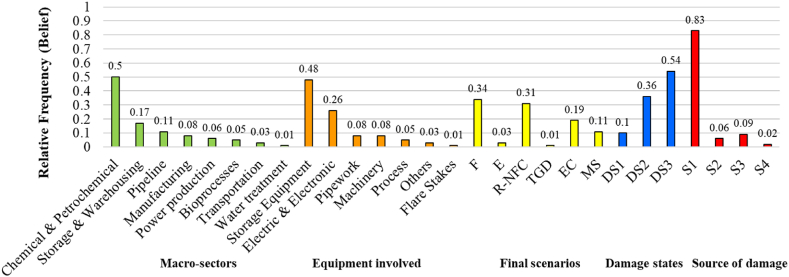
Relative frequencies (belief) for the five functional variables considered in the model, after treating the uncertainty for the unknown records.

Additional information about the conditional probabilities of the key variable “equipment,” given the other variables, could be extracted from the next Table 9. On the other hand, Fig. 15 offers the relative frequencies for the different industrial attributes after the mathematical treatment of the missing values in the dataset.

Regarding Table 9 and Fig. 15, it is not our purpose to discuss in detail the results they have shown, which are consistent with the flow so far described. On the other hand, the novelty lies in the innovative insights that the methodology here developed enables, thereby expanding the understanding of vulnerability to lightning strikes. These novel insights offer a new probabilistic meaning to the uncertain historical data that otherwise remained in the dark, providing an important additional view that contributes to strengthening the decision-making process to face the industrial vulnerability against lightning strikes.

**Table 9:** CPTs for Equipment involved/Damage states and Source of structural damage (extended dataset).

Macro-sector		DS1	DS2	DS3	S1	S2	S3	S4
**Equipment**	**Process**	0.08	0.03	0.06	0.05	0.03	0.07	0.01
	**Storage Equipment**	0.78	0.72	0.27	0.55	0.24	0.14	0.09
	**Pipework**	0.03	0.08	0.09	0.08	0.18	0.06	0.04
	**Electric & Electronic**	0.03	0.1	0.4	0.23	0.29	0.39	0.65
	**Machinery**	0.02	0.02	0.13	0.05	0.14	0.25	0.16
	**Flare Stakes**	0	0.01	0.01	0.01	0.04	0.04	0.01
	**Others**	0.05	0.04	0.03	0.03	0.07	0.04	0.04

## 4 Conclusions and implications

A lightning-triggered NaTech-driven dataset has been compiled with 689 records. This included a meticulous analysis of past events registered on prominent open-source databases. The research demonstrates a clear link between the number of records gathered within specific geographic areas and the source nationality of the consulted databases. Additionally, the recurrent presence of the “unknown” category evidenced the uncertain and insufficient information registered. Indeed, this issue limits the possibility of learning from past events, highlighting not only the critical need for a systematic gathering and standardization of information about NaTech events on a global scale but also the necessity of implementing modeling methods to identify hidden signals within the current data that trigger complex scenarios.

The trend of records over the years is marked by substantial variability. Since 2010, there has been an observed subtle decline in the average count of recorded events, reflecting a trend consistent across all databases. Further investigation warrants exploration into the interplay between the lifespan of events triggered by lightning, the temporal evolution of lightning protection standards, and the regulatory scenery governing event reporting across the principal geographical regions under scrutiny.

Approximately 83.3 % of these NaTech events took place during the spring and summer seasons. This observed pattern of seasonality intersects conceptual ideas in the manufacturing field such as inspections and audits for proactive planning. The seasonality of lightning was contextualized within the current Italian inspection plans for Seveso establishments, analyzing the 2017 inspection plan for upper-tier establishments as a punctual example. It shows that 54 % of the 161 inspections for 2017 were programmed during the spring and summer seasons. Consequently, the corrective actions that could be detected associated with the lightning protection system probably will not be implemented in time to avoid the most critical seasons for the lightning aspect. Additional research should be conducted to further elaborate on these conceptual ideas, including the territorial implications associated with the intensity of the natural hazard of concern.

Incidents and loss of containment together account for more than 80 % of the 689 records retrieved for NaTech events triggered by lightning strikes in the process industry. Nearly all the events analyzed took place during the normal operations of the plant. Once again, the limited information available in the records regarding safety barriers stands as a stark warning that increased attention must be directed toward this critical concern.

Regarding the vulnerable industrial macro-sectors, chemical and petrochemical, storage and warehousing, pipelines, and manufacturing contributed to around 85 % of the documented cases. In contrast, the remaining categories, including power production, bioprocesses, transportation, and water treatment, constituted the remaining 15 %. The presence of 283 records without classification limits the certainty of the frequency of this industrial attribute.

Storage equipment emerged as the most susceptible industrial item, representing approximately 40 % of certain occurrences, while electrical equipment and electronic devices followed closely at 21 %. Specifically in the sectors chemical and petrochemical, storage and warehousing, transportation, and bioprocess, the storage equipment prevalence accounts for proportions ranging between 0.43 and 0.85 of recorded events in the dataset. Approximately 15 % of the records lack the necessary information to classify the vulnerable equipment.

As a consequence of the lack of valuable information for the principal source of data, the technological scenarios triggered were characterized using a refined subset of 127 observations, including all the “other sources” of data. The fire was the most prominent scenario observed, accounting in general for approximately 56 % of the analyzed subset, followed by multiple scenarios (21.3 %), which were particularly pronounced when the primary scenario was an explosion (80 % of the time). The collective trend commented on above underscores that scenarios involving ignition—whether single or multiple, immediate or delayed—comprise slightly over 86 % of the total data in the subset. Consequently, the substances classified as physical hazards accumulated around 70 % of the total triggered scenarios, with a substantial 92 % attributed to flammable substances, of which 63 % are associated with different types of hydrocarbons. Despite some records lacking sufficient information to assess the consequences of this kind of NaTech event, the quantified losses underline that most of them fulfill the requirements to be categorized as major accidents, even if, in several cases, these losses occur in industrial plants that are not categorized as major accident plants.

The logic pathways for the events leading to final ignition scenarios (107 records) were linked to the lightning source of structural damage and the damage state as functional intermedial attributes between the initiating event and the final consequences. Among the potential branches in the event tree, the direct pathway (lightning impacting directly the industrial structure) was the primary cause of collapses and severe damages in the documented cases. This path accounted for 36 % of the classifiable records that directly affected the structure, while more than 50 % of them did not. Although the indirect pathway has shown lower frequency in historical incidents, it is important to consider this approach within the industrial lightning protection system and protective measures for mitigation of NaTech triggered by lightning.

The implementation of Bayesian networks represented a robust methodological framework to face the current limitations affecting the available information in open-source industrial accident databases. It made it possible to get conditional probabilities from the event tree and improved the model by adding attributes for vulnerable equipment and macro-sectors. This expansion revealed that “equipment involved” emerged as the pivotal variable in the model governing conditional probabilities on critical dependencies associated with the source of damages, infrastructural damage state, final scenario, or industrial macro-sector.

The implemented mathematical algorithms provide a significant secondary perspective for unclassifiable data within the lightning-triggered NaTech-driven dataset, shedding light on what was previously unclear. For example, the updated belief frequency regarding the direct impact of lightning on structures increased from 36 % to 83 % after addressing unknown data. Furthermore, the identification of storage equipment as a critical vulnerable item rose from 40 % to 48 %, and the chemical and petrochemical macro-sector increased from 29 % with certain data to 50 % after addressing uncertainty. This dual perspective on the data, both before and after handling unknown records, offers objective insights that serve as an initial step in addressing vulnerabilities and increasing awareness, contingent upon the assumed level of confidence.

The research approach presented here makes a significant methodological contribution that can be applied broadly to tackle the impact of different NaTech factors on industrial facilities within the context of the multiple hazards facing critical infrastructure. Equally important is consideration for local stakeholders. By pinpointing industrial vulnerability to lightning strikes, these findings can be linked with meteorological data related to the plants geographical location and the intensity of such natural disasters, like ground lightning density (Ng). Subsequent research have been initiated to bridge the gap between the operational vulnerabilities of industrial plants and those linked to their specific geographical location (the function-location approach).

## Funding

Part of this study was carried out within the RETURN Extended Partnership and received funding from the European Union Next-GenerationEU (National Recovery and Resilience Plan – NRRP, Mission 4, Component 2, Investment 1.3 – D.D. 1243 August 2, 2022, PE0000005). Likewise, part of this work has been done within the Collaborative Intelligence for Safety-Critical Systems project (CISC). The CISC project has received funding from the European Union Horizon 2020 Research and Innovation Programme under the Marie Skłodowska-Curie grant agreement no.955901.

## Data availability statement

The data associated with this study has been deposited into the publicly available repository Mendeley Data. DOI: https://doi.org/10.17632/fff64w3rzn.3.

## Ethics statement

This research did not involve any applicable ethics statement and all research procedures were carried out in accordance with the requirements for ethical principles.

## CRediT authorship contribution statement

**David Javier Castro Rodriguez:** Writing – original draft, Visualization, Software, Methodology, Investigation, Formal analysis, Data curation, Conceptualization. **Joseph Mietkiewicz:** Visualization, Software, Methodology, Data curation. **Morena Vitale:** Visualization, Investigation, Data curation. **Gabriele Baldissone:** Validation, Supervision, Formal analysis. **Antonello A. Barresi:** Writing – review & editing, Validation, Supervision, Project administration, Funding acquisition, Formal analysis. **Micaela Demichela:** Writing – review & editing, Validation, Supervision, Project administration, Methodology, Conceptualization.

## Declaration of competing interest

The authors declare that they have no known competing financial interests or personal relationships that could have appeared to influence the work reported in this paper.

## Footnotes

1 Legislative Decree No. 105 of June 26, of 2015 “Implementation of Directive 2012/18/EU on the control of major-accident hazards involving dangerous substances”. Italian Official Gazette General Series No. 161 14/07/2015-Suppl. Ordinary No. 38.

2 Note from the Department of Fire, Public Rescue and Civil Defense, Central Directorate for Prevention and Technical Safety of the Ministry of the Interior, note prot. no.1834 of Feb. 12, 2016, “Legislative Decree 105/2015. National plan of inspections”. dipvvf. DCPREV. Official Register U. 0001834.12-02-2016.
